# Acute multifocal hemorrhagic retinal vasculitis in a child: a case report

**DOI:** 10.1186/s12886-016-0360-8

**Published:** 2016-10-18

**Authors:** Malik Y. Ghannam, Mohammed Naseemuddin, Peter Weiser, John O. Mason

**Affiliations:** 1 AN-Najah University Teaching Hospital, Asira, Nablus, West Bank Palestine; 2 University of Alabama (UAB), Birmingham, AL USA; 3 Clinical Immunology and Rheumatology, University of Alabama (UAB), Birmingham, AL USA

**Keywords:** Acute multifocal hemorrhagic retinal vasculitis, Corticosteroids, Cyclophosphamide, Mycophenolate, Rituximab, Case report

## Abstract

**Background:**

Acute Multifocal Hemorrhagic Retinal Vasculitis (AMHRV) is a rare disease with unknown incidence that presents with abrupt onset of visual loss associated with retinal vasculitis, retinal hemorrhage, non-confluent posterior retinal infiltrates, vitreous cellular inflammation and papillitis in, otherwise, healthy adult individuals. The reported treatment options for Acute Multifocal Hemorrhagic Retinal Vasculitis are oral corticosteroids, intravitreal ganciclovir and laser photocoagulation or vitrectomy. We report a child with Acute Multifocal Hemorrhagic Retinal Vasculitis who was treated with aggressive immunosuppressive therapy resulting in a favorable visual outcome.

**Case presentation:**

A retrospective case report of a 10-year-old African American girl who developed unilateral Acute Multifocal Hemorrhagic Retinal Vasculitis, which later on progressed bilaterally. We conducted a review of the clinical, laboratory and photographic records to evaluate her functional and anatomic outcome after aggressive immunosuppressive treatment. During the first 4 months of treatment of OD with intravitreal ganciclovir, intravitreal dexamethasone and systemic prednisone, the change in vision in OD improved from light perception (LP) to counting fingers (CF). During the next 18 months of aggressive systemic treatment of OD and the newly affected left eye (OS), the change in vision improved from CF in OD and CF in OS to 20/200 in OD and 20/80 in OS. Management during the 18-month interval included rituximab infusions, cyclophosphamide/methylprednisolone infusions, prednisone and mycophenolate.

**Conclusions:**

This is the first reported case of Acute Multifocal Hemorrhagic Retinal Vasculitis occurring in a child. Ophthalmologists should be aware of the need to treat severe Acute Multifocal Hemorrhagic Retinal Vasculitis with aggressive immunosuppressive agents in collaboration with rheumatologists to obtain the best possible visual outcome.

## Background

Acute Multifocal Hemorrhagic Retinal Vasculitis (AMHRV) is a rare disease with unknown incidence. It was first reported by Blumenkranz et al. in 1988 as an episodic disease that presents with abrupt onset of visual loss associated with retinal vasculitis, retinal hemorrhage, non-confluent posterior retinal infiltrates, vitreous cellular inflammation and papillitis in, otherwise, healthy adult individuals [1]. Although, the exact etiology of this disease is not known, many of the features of AMHRV may resemble those of an acute viral infection and some autoimmune diseases especially Behcet’s disease [1]. Consequently, it is often treated, initially, with anti-viral medications without any improvement. Other conditions with similar ocular findings can mimic AMHRV including Behcet’s disease, Eales disease, systemic lupus erythematosus, ocular syphilis, viral disease and sarcoidosis.

Once the diagnosis of AMHRV is clinically established, treatment should begin immediately to avoid complications including epiretinal membranes, retinal neovascularization, iris neovascularization, vitreous hemorrhage, optic neuropathy or neovascular glaucoma [1, 2] resulting in visual loss. Currently, treatment options for AMHRV have included laser photocoagulation, oral corticosteroids, vitrectomy and intravitreal ganciclovir [1–3].

To date, only adults have been described in the literature to develop AMHRV. Herein, we report a child who developed AMHRV in her right eye (OD) followed by left eye (OS) involvement 4 months later, treated with aggressive immunosuppressive therapy that included the use of rituximab, cyclophosphamide, methylprednisolone, prednisone and mycophenolate, which resulted in remarkable improvement in visual acuity. We believe patients with severe cases of AMHRV may benefit from early and aggressive immunosuppressive therapy.

## Case presentation

**Fig. 1:**
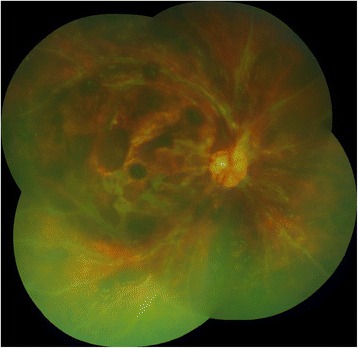
Color photograph of right eye showing severe intraretinal hemorrhages

A 10-year-old African-American girl with a history of asthma and eczema had 20/20 vision in both eyes until August 8, 2011, when she first noticed floaters in her OD. On August 10, 2011, the patient developed abrupt onset of visual loss in the OD. She presented to the emergency department (ED), complaining of unilateral visual loss in the OD. No other systemic complaints were reported. Patient was found to have visual acuity of light perception (LP) in the OD and 20/20 in the OS. Results of a slit-lamp examination of the OD showed clear cornea, trace conjunctival injection and a few cells in the anterior chamber. On fundoscopic exam of the OD, patient was found to have 1+ cells in the anterior vitreous as well as severe intraretinal hemorrhage, exudates, perivasculitis and posterior vitritis. The optic nerve could not be visualized (Fig. 1). Examination of the OS did not reveal any abnormalities. Intraocular pressures in both eyes were within normal limits.

**Fig. 2:**
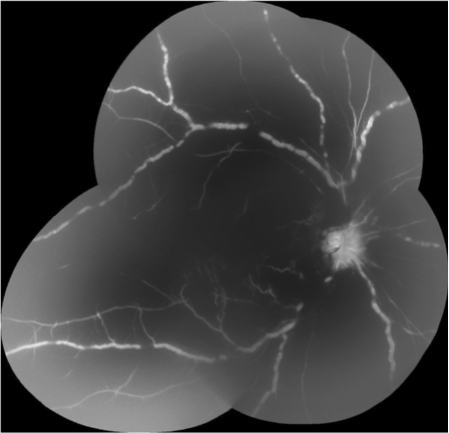
Fluorescein angiography of the right eye showing severe blocking defects from intraretinal hemorrhage and retinal vasculitis

Chest x-ray and MRI of the brain did not reveal any abnormalities. Laboratory examination included complete blood count, complete metabolic profile, urinalysis, FTA-abs, human immunodeficiency virus (HIV) panel, and serology for varicella zoster virus (VZV), herpes simplex virus (HSV) I/II and toxoplasmosis. Anterior chamber tap was also done to test the fluid for VZV, HSV I/II and cytomegalovirus DNA through polymerase chain reaction (PCR). We also tested the patient for ANCA associated vasculitides, rheumatoid arthritis factor (RF), ANA panel, erythrocyte sedimentation rate (ESR) and angiotensin converting enzyme levels to evaluate for a non-infectious cause. All of the tests were unremarkable except for: elevated ESR at 32 mm/h (normal: <20), elevated ACE levels at 124 U/L (normal: 6–89) and elevated eosinophils at 8.5 % (normal: 1–5 %). Fluorescein angiography (FA) revealed vasculitic process with intraretinal hemorrhage blocking defects and there was retinal ischemic changes (Fig. 2).

**Fig. 3:**
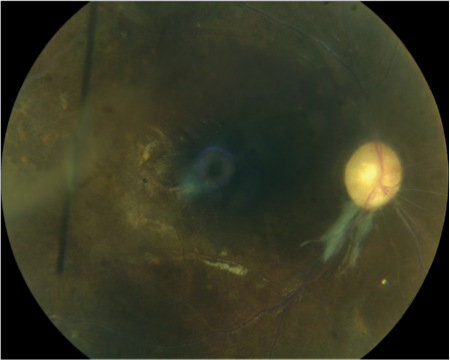
Color photograph of right eye following resolution of AMHRV showing optic nerve pallor and narrowed retinal vessels

Patient was placed on long-term therapy with famcyclovir 500 mg for a presumed viral infection. We gave one mg gancyclovir three times 5 days apart each with 400 microgram dexamethasone intravitreal injections in OD starting on August 15, 2011. In addition to the injections, patient was started on oral Prednisone 50 mg daily. Over the next few weeks, retinal vasculitis continued to improve OD but her visual acuity remained poor at counting fingers (CF). Fundoscopic exam of the OD on December 1, 2011, revealed some optic nerve pallor with ischemic and narrowed vessels with resolution of retinal hemorrhage (Fig. 3).

**Fig. 4:**
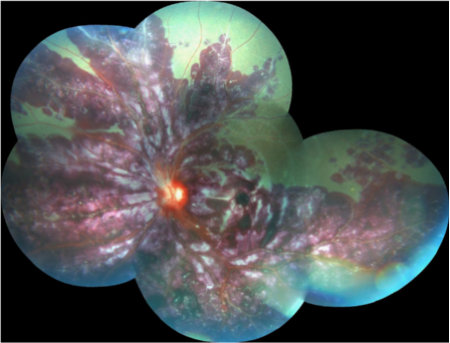
Color photograph of left eye showing severe intraretinal hemorrhage and vasculitis

While the OD continued to stabilize, the AMHRV progressed to involve the OS. Patient reported decreased visual acuity in the OS starting November 29, 2011. Her visual acuity continued to deteriorate over the next few days and at the time of exam on December 1, 2011; patient’s visual acuity was CF. On fundoscopic exam, she was found to have severe intraretinal hemorrhage with retinal vein sheathing and whitening (Fig. 4). Rheumatology was consulted. Per rheumatology recommendations, patient was admitted to the hospital for further evaluation and aggressive treatment. Patient underwent the prior mentioned laboratory evaluation again. In addition to the previous laboratory tests, we obtained serum lysozyme levels, IgG/IgM levels, CH50 levels and A-1-C enzyme levels. The work-up was unremarkable except for elevated eosinophils at 8.8 % (normal: 1–5 %), elevated sedimentation rate of 70 mm/h (normal: 0–20 mm/h) and low CH50 of 27 U/mL (normal: 31U/mL-60U/mL). On this occasion, her ACE levels were within normal limits.

**Fig. 5:**
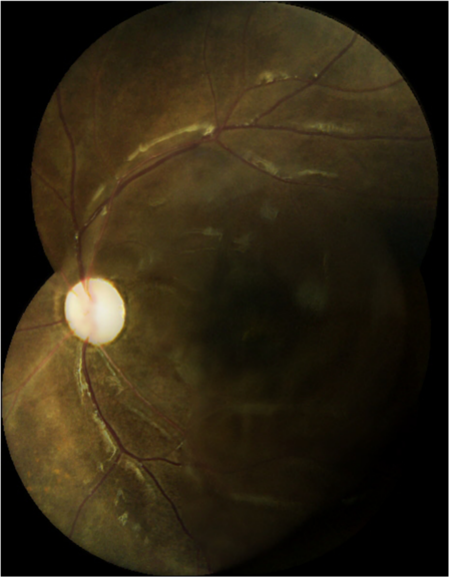
Color photograph of left eye following resolution of AMHRV

Due to her poor vision of CF OD and new onset AMHRV OS with CF vision, she was aggressively treated with three infusions of rituximab (650 mg/m^2^), two of which were in December 2011 and the third in June 2012. She also received alternating six cycles of cyclophosphamide (1 g/m^2^)/methylprednisolone 500 mg (1 g/m^2^) intravenous infusions every 4 weeks from December 2011-June 2012. In addition to the infusions, patient was placed on Prednisone 60 mg a day that was slowly tapered over 7 months. B-cell depletion was monitored through flow cytometry. After the completion of the infusions and steroid taper, patient was placed on mycophenolate 1000 mg BID to prevent recurrence of retinal vasculitis. Patient’s visual acuity in the OS improved from CF to 20/80 within 1 month of initiation of aggressive immunosuppressive treatment and has remained stable. On the most recent fundoscopic exam of the OS in June 2013, the optic nerve appeared healthy without any evidence of vasculitis, hemorrhage or phlebitis (Fig. 5). Although there was no ischemic changes at the onset of the disease, retinal ischemia developed later in FA during follow up. Functional improvement was also noted in the OD during the aggressive immunosuppressive course of therapy, resulting in visual improvement from CF to 20/200.

## Discussion

We report a case of AMHRV in a 10-year-old girl who presented with abrupt onset of visual loss secondary to severe retinal hemorrhage and vasculitis. On fundoscopic exam, we noticed retinal arteritis and phlebitis with sheathing. Her medical history was unremarkable except for a prior diagnosis of asthma and eczema. The disease process in our patient was subsequently bilateral, with the OS developing the disease 4 months after the OD. Thorough systemic medical evaluation to determine the etiology of the retinal vasculitis was unremarkable except for elevated ESR and eosinophils. Consequently, the diagnosis of AMHRV was made on clinical examination.

AMHRV diagnosis is established mainly clinically, Table 1 summarizes the diagnostic criteria of AMHRV based on Blumenkranz. Although It is a clear according to the literature that there is a relation between autoimmune diseases such as bronchial asthma and AMHRV which was noticed in Blumenkrank cases and our case as well, the exact pathophysiology of acute multifocal hemorrhagic retinal vasculitis is still unknown [1]. It is probably an unknown viral etiology. Later on, the most common ocular complications of AMHRV arise from the retinal ischemic changes. These complications includes: epidural membrane formation, neovascular glaucoma, retinal detachment and vitreous hemorrhage [1]. Nothing of the aforementioned problems were noticed in our patient, since it was newly diagnosed and treated.

**Table 1:** Clinical criteria for diagnosis of AMHRV

Abrupt onset of unilateral or bilateral visual loss	
Predominant venular retinal vaculitis	
Variable retinal hemorrhages	
Nonconfluent posterior retinal infiltrates	
Vitreous cellular inflammation	
Papillitis	

A systemic inflammatory disease like Behcet’s disease may present with similar fundus manifestations and clinical course as our patient. Behcet’s disease is common in regions along the Silk Road, from the countries of the Mediterranean to the Far East. It commonly affects males who are 20–40 years old; however, 26 % of individuals with Behcet’s disease are younger than 16 years of age [4]. Based on the new International Criteria for Behçet’s Disease, a patient scoring ≥ 4 points is classified as having Behcet’s disease. Ocular lesions, oral aphthosis and genital aphthosis are each assigned 2 points, while skin lesions, central nervous system involvement and vascular manifestations, 1 point each. The pathergy test, when used, was assigned 1 point. [5]. Our patient does not fit the aforementioned criteria for Behcet’s disease.

Eales disease presents with fundoscopic findings that are similar to those found in AMHRV. Eales disease is an idiopathic inflammatory disease, typically affecting young men, that is characterized by retinal phlebitis, peripheral non-perfusion and retinal neovascularization [6]. Patients often present with recurrent vitreous hemorrhages secondary to retinal neovascularization. Our patient does not fit the typical patient demographic for Eales disease and at no point did she develop vitreous hemorrhages during her 22-month follow-up. In addition, Eales disease does not usually present with 360° of retinal hemorrhage, as was the case with our patient.

Viral retinopathies secondary to herpes class viruses can mimic the fundoscopic findings of AMHRV. The presentation can vary from acute retinal necrosis (ARN) in immunocompetent patient to progressive outer retinal necrosis (PORN) in an immunocompromised individual [7]. Vasculitis caused due to a viral infection typically involves the arterioles [7], whereas AMHRV involves the retinal venules. In addition, PCR was performed on the anterior chamber fluid and on the vitreous sample obtained from our patient, which showed no evidence of a herpetic viral process.

Due to the rare presentation of AMHRV, there have been no controlled trials conducted to determine a definitive treatment protocol for this disease. A thorough review of the literature yields nine published cases of AMHRV and the treatments that were utilized. All patients were adults at the time of presentation, while this report is the only known case of AMHRV developing in a child. Blumenkranz et al. reported a series of seven patients who were treated using oral corticosteroids, azathioprine, panretinal photocoagulation, acyclovir and anticoagulants [1]. In a case report from Brazil, Amaro et al. reported improved visual outcome over the long-term using panretinal photocoagulation and oral corticosteroids [2]. Bronner et al. reported using intravitreal ganciclovir, oral corticosteroids, panretinal photocoagulation and vitrectomy to treat their patient with AMHRV [3]. Our management, during the treatment of the OD, included intravitreal ganciclovir, intravitreal dexamethasone, famcyclovir and prednisone. Although the treatment was initiated within a week of symptom onset, it did not result in a significant improvement in visual acuity. It was, however, beneficial in improving retinal hemorrhage and vasculitis. Our decision to use famcyclovir instead of acyclovir was based on published studies that illustrated the efficacy of this drug in treating acute retinal necrosis [8]. Our treatment regimen was significantly altered when the patient developed AMHRV involving the OS. After rheumatology consultation, we aggressively treated with potent immunosuppressants considering her poor visual prognosis. Patient was treated with rituximab infusions, cyclophosphamide/methylprednisolone infusions, oral prednisone and mycophenolate. Our decision to use rituximab and mycophenolate was based on published studies that illustrated the efficacy of these drugs in treating retinal vasculitis. Davatchi et al. and Donnithorne et al. have reported using rituximab infusions for the treatment of refractory retinal vasculitis secondary to Behcet’s disease and SLE, respectively [9, 10]. In a retrospective cohort study with 257 patients, Galor et al. found that mycophenolate relieved inflammation significantly faster than methotrexate, while causing fewer adverse events than azathioprine [11]. We, in conjunction with rheumatology, carefully monitored the patient for the development of side effects. Patient did develop mild neutropenia during the course of her treatment, but remained asymptomatic throughout the 22-month follow-up. Only one month after the initiation of treatment, which included two cycles of rituximab infusions, one cycle of cyclophosphamide/methylprednisolone infusion and oral prednisone; we noted a remarkable improvement in patient’s visual acuity in the OS. It improved from CF to 20/80 over a 1 month period and this improvement was sustained throughout the 22-month follow-up. The OD, only after initiation of aggressive immunosuppressive therapy, slowly regained vision from CF to 20/200. Donnithorne et al. reported a similar experience in treating two pediatric cases with SLE induced retinal vasculitis, where delay in treatment resulted in limited visual recovery [10].

## Conclusions

We present the first case of AMHRV developing in a child. Early treatment with potent immunosuppressants like rituximab, cyclophosphamide, methylprednisolone, prednisone and mycophenolate may not only reduce retinal hemorrhagic vasculitis, but also result in remarkable improvements in visual outcome.

## Abbreviations

AMHRV: Acute Multifocal Hemorrhagic Retinal Vasculitis
ARN: Acute retinal necrosis
BID: Bis in die (twice a day)
CF: Counting fingers
ESR: Erythrocyte sedimentation rate
FA: Fluorescein angiography
HIV: Human immunodeficiency virus
HSV: Herpes simplex virus
LP: Light perception
OD: Oculus dexter
OS: Oculus sinister
PCR: Polymerase chain reaction
PORN: Progressive outer retinal necrosis
RF: Rheumatoid arthritis factor
SLE: Systemic lupus erythrematosus
VZV: Varicella zoster virus
